# Pulsed Field Ablation for Atrial Fibrillation: Mechanism, Clinical Evidence, and Emerging Applications

**DOI:** 10.7759/cureus.115160

**Published:** 2026-08-25

**Authors:** Kyrillos Girgis, Mena Elmalh, Tony Elias, Howard Levitt, Shawn Lee, Sergio Waxman, David Man

**Affiliations:** 1 Cardiology, Newark Beth Israel Medical Center, Newark, USA; 2 Internal Medicine, Newark Beth Israel Medical Center, Newark, USA; 3 Internal Medicine, Temple University, Philadelphia, USA; 4 Electrophysiology, Newark Beth Israel Medical Center, Newark, USA

**Keywords:** atrial fibrillation, cardiac catheter ablation, irreversible electroporation, pulmonary vein isolation, pulsed field ablation

## Abstract

Pulsed field ablation (PFA) is a nonthermal catheter ablation modality that uses high-voltage, ultra-short electrical pulses to induce irreversible electroporation of cardiomyocyte membranes. Unlike radiofrequency and cryoballoon energy, PFA exploits differential electroporation thresholds across tissue types, producing lesions that are largely confined to the myocardium while sparing the esophagus, phrenic nerve, and pulmonary veins. Multiple pivotal trials and large multinational registries have now established PFA as an effective and safe alternative to thermal ablation for both paroxysmal and persistent atrial fibrillation (AF), with freedom from atrial arrhythmia rates comparable to radiofrequency and cryoablation, and a distinct, energy-specific complication profile. Notable adverse events unique to PFA include transient coronary artery vasospasm, intravascular hemolysis with rare acute kidney injury, and microbubble-related embolic risk, while classic thermal complications, such as atrioesophageal fistula and pulmonary vein stenosis, have shown a markedly reduced observed incidence across large cohorts. This review summarizes the biophysical mechanism of PFA, synthesizes clinical trial and registry evidence for paroxysmal and persistent AF, details the energy-specific safety profile, and outlines emerging applications, including posterior wall isolation, ventricular arrhythmia ablation, and next-generation catheter design. As PFA adoption accelerates, understanding both its favorable safety profile and its unique failure modes is essential for electrophysiologists integrating this technology into practice.

## Introduction and background

Catheter ablation is a cornerstone therapy for symptomatic atrial fibrillation (AF) refractory to antiarrhythmic drug therapy, with pulmonary vein isolation (PVI) as its procedural foundation. For nearly three decades, PVI has relied on thermal energy sources, namely, radiofrequency (RF) ablation and cryoballoon ablation, which create lesions through coagulation necrosis or cellular freezing, respectively. RF ablation delivers point-by-point lesions using resistive heating from an electrode tip, while cryoballoon ablation delivers a single-shot circumferential freeze around each pulmonary vein; both approaches have been refined over decades of clinical use, but their reliance on generating extremes of temperature to destroy tissue has not changed. Both modalities are effective but carry a well-documented, if uncommon, risk of collateral thermal injury to structures adjacent to the left atrium, including the esophagus (atrioesophageal fistula), phrenic nerve (persistent palsy), and pulmonary veins (stenosis) [1].

Pulsed field ablation (PFA) represents a fundamentally different energy paradigm. Rather than heating or freezing tissue, PFA delivers microsecond-to-millisecond high-voltage electrical pulses that disrupt the lipid bilayer of the cardiomyocyte membrane through irreversible electroporation (IRE), triggering cell death without generating clinically significant heat [2]. In simple terms, the electrical pulse acts like a targeted switch that punches short-lived, then permanent, holes in the outer membrane of the heart muscle cell, causing it to die without the surrounding heat or cold that damages nearby tissue with older techniques. Because the field intensity required to electroporate cardiomyocytes is substantially lower than that required for many surrounding tissues, PFA offers a theoretical degree of tissue selectivity, meaning it can disable heart muscle while leaving less-susceptible neighboring structures largely unharmed, that thermal energy cannot replicate [2]. Following earlier first-in-human experience and a wave of U.S. regulatory approvals spanning late 2023 through 2024, PFA has moved rapidly from investigational novelty to a mainstream ablation strategy at high-volume centers worldwide [1,3]. This review examines the mechanistic basis of PFA, summarizes the pivotal trial and real-world registry evidence, characterizes its distinct safety profile, and discusses emerging indications beyond standard pulmonary vein isolation.

This narrative review is based on a targeted search of PubMed/MEDLINE and a manual review of major cardiology journals (the New England Journal of Medicine, Circulation, the Journal of the American College of Cardiology, Heart Rhythm, Nature Medicine, and EP Europace) for pivotal randomized trials, large multinational registries, and systematic reviews or meta-analyses on pulsed field ablation published through August 2026. Formal risk-of-bias assessment and quantitative synthesis were not performed, in keeping with the narrative format of this review.

Mechanism of action: irreversible electroporation

**Figure 1:**
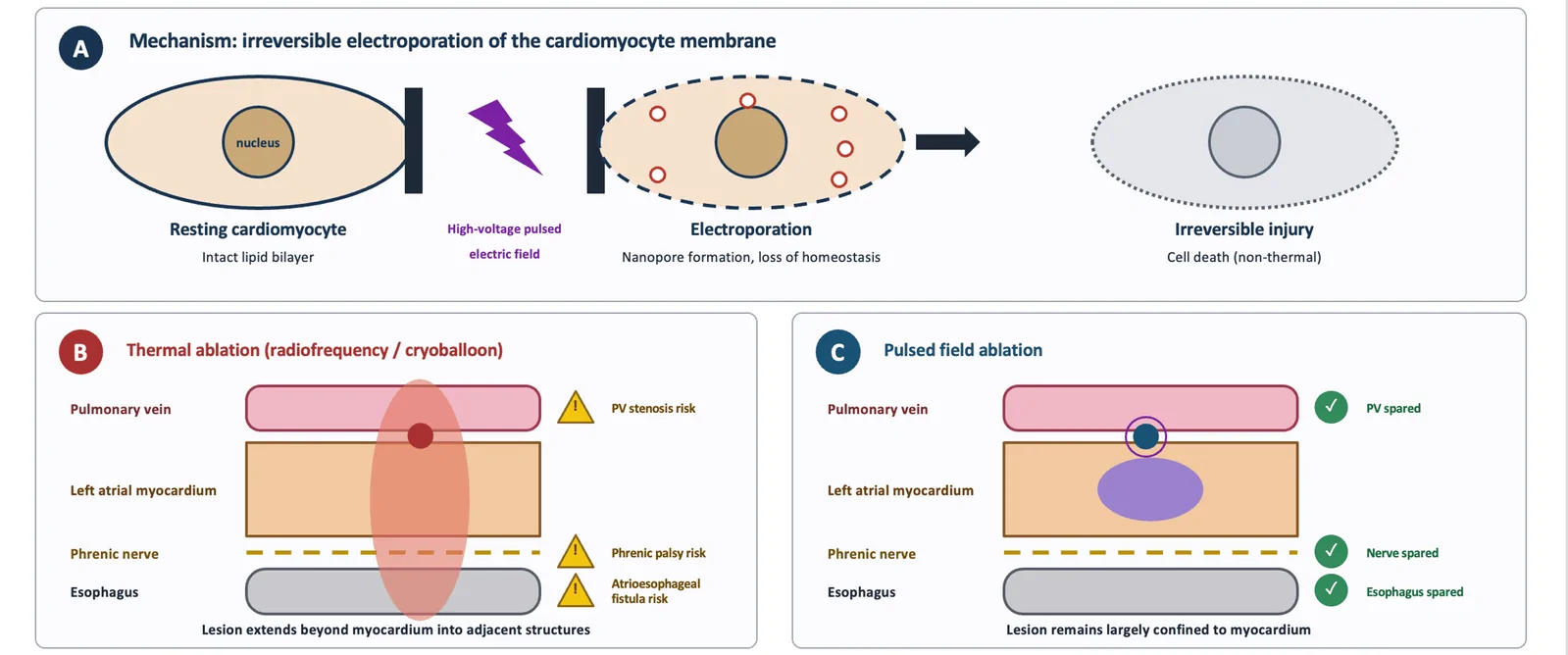
Mechanism of pulsed field ablation and tissue-selective lesion formation (A) A high-voltage pulsed electric field applied across the cardiomyocyte membrane produces nanoscale pores (electroporation); above a critical field strength, this injury becomes irreversible, and the cell dies through a nonthermal mechanism. (B) With thermal energy sources (radiofrequency or cryoballoon), the ablation lesion can extend beyond the myocardium into adjacent structures, creating risk for pulmonary vein (PV) stenosis, phrenic nerve palsy, and atrioesophageal fistula. (C) Because myocardium electroporates at lower field intensities than surrounding tissue, the PFA lesion remains largely confined to the myocardial wall, sparing the adjacent PV, phrenic nerve, and esophagus. PV, pulmonary vein. Figure created by the authors using Microsoft PowerPoint (Microsoft Corporation, Redmond, WA, US), based on data from Reddy et al. (ADVENT) [1], Chinyere et al. [2], Ranjbartehrani [4], Turagam et al. (MANIFEST-PF) [5], and Ekanem et al. (MANIFEST-17K) [6].

Electroporation occurs when an externally applied electric field exceeds the transmembrane potential threshold of a cell, opening nanoscale pores in the lipid bilayer. At lower field strengths, this process is reversible and forms the basis of gene and drug delivery techniques. Above a critical threshold, however, pore formation becomes irreversible, disrupting ionic homeostasis and leading to cell death; this is the mechanism PFA exploits therapeutically [2,4] (Figure 1). 

Cardiomyocytes reach the irreversible electroporation threshold at comparatively low field intensities relative to vascular smooth muscle, fibroblasts, red blood cells, and neural tissue [2]. This differential susceptibility underlies the tissue-selective claim of PFA: at appropriately calibrated voltages, myocardial tissue can be ablated while adjacent non-myocardial structures, most notably the esophagus and phrenic nerve, are relatively spared. Preclinical and clinical data have supported this selectivity, with large registries reporting a markedly reduced observed incidence of esophageal thermal injury or pulmonary vein stenosis relative to thermal energy sources [5,6], complications that remain a persistent, if infrequent, concern with RF and cryoballoon energy. However, tissue selectivity is relative rather than absolute, and the field is not entirely without collateral effects, as detailed further on in this article [2,4].

## Review

Clinical evidence

Paroxysmal Atrial Fibrillation

The ADVENT trial was the first randomized comparison of the Farapulse pulsed field ablation system (Boston Scientific, Marlborough, MA, US) against conventional thermal ablation (RF or cryoballoon) in patients with drug-refractory paroxysmal AF [1]. Among 607 randomized patients, 12-month freedom from atrial arrhythmia was 73.3% with PFA versus 71.3% with thermal ablation (between-group difference 2.0 percentage points, 95% Bayesian credible interval -5.2 to 9.2), meeting prespecified noninferiority criteria (margin 15 percentage points) for both safety and effectiveness [1]. PFA was associated with shorter total procedure and ablation times, though fluoroscopy time was longer, and pulmonary vein narrowing was less frequent than with thermal energy [1]. A subsequent substudy using weekly transtelephonic monitoring and serial Holter recordings found that PFA-treated patients had a statistically significantly lower residual atrial arrhythmia burden than those treated with thermal energy [1].

**Figure 2:**
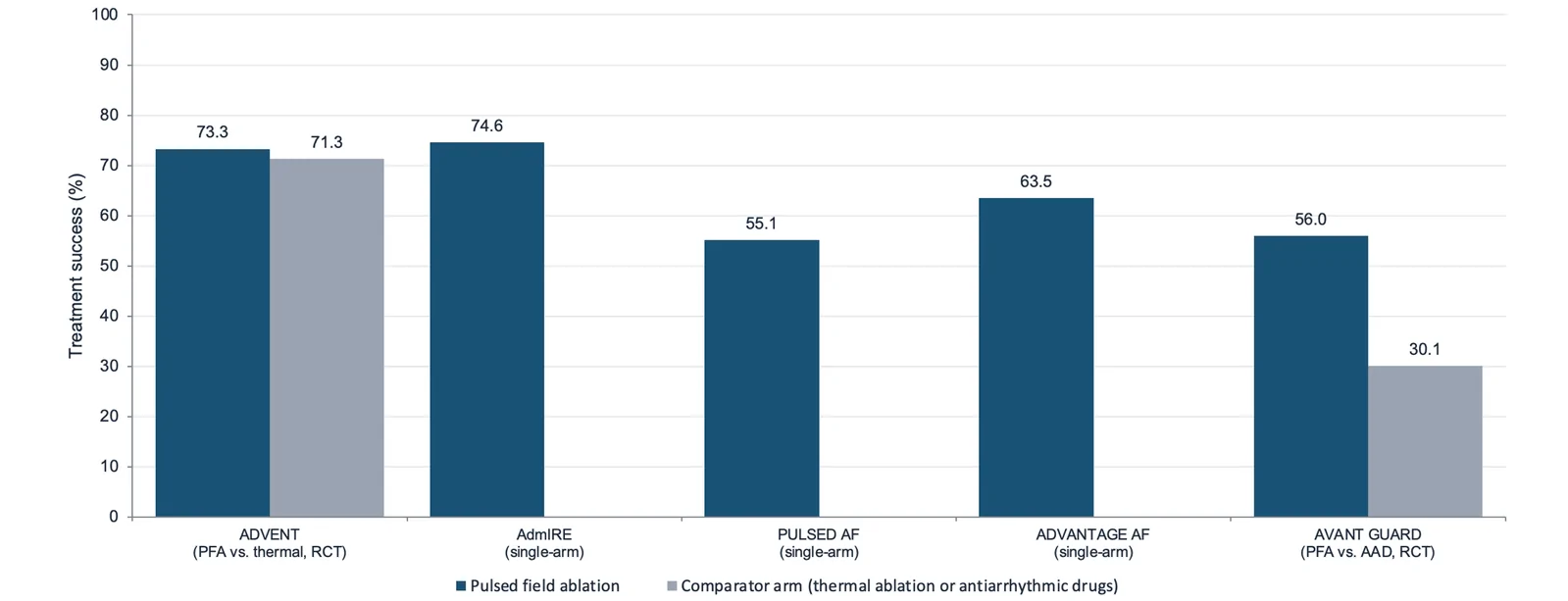
Primary effectiveness at approximately 12 months across major PFA trials ADVENT and AVANT GUARD were RCTs comparing PFA against an active comparator (thermal ablation and antiarrhythmic drug (AAD) therapy, respectively); AdmIRE, PULSED AF, and ADVANTAGE AF were single-arm trials evaluated against prespecified performance goals. Endpoint definitions vary by trial (composite freedom from arrhythmia, acute failure, or antiarrhythmic escalation); see manuscript text for exact definitions. These trials differ in design, population, PFA platform, and endpoint definition; the values shown should not be read as direct comparisons of relative efficacy across trials or platforms. PFA, pulsed field ablation; RCT, randomized controlled trial; AAD, antiarrhythmic drug Figure created by the authors using data from Reddy et al. (ADVENT) [1], Verma et al. (PULSED AF) [3], Reddy et al. (AdmIRE) [7], Reddy et al. (ADVANTAGE AF) [9], and Wazni et al. (AVANT GUARD) [10].

The single-arm PULSED AF (Pulsed Field Ablation to Irreversibly Electroporate Tissue and Treat AF) pivotal trial, evaluating the PulseSelect system (Medtronic, Minneapolis, MN) in 150 paroxysmal and 150 persistent AF patients across 41 centers, reported a low primary safety adverse event rate (0.7%) and acute pulmonary vein isolation in all cases, supporting the initial regulatory approvals for PFA in the United States [3]. The AdmIRE (Assessment of Safety and Effectiveness in Treatment Management of Atrial Fibrillation With the Bosense-Webster Irreversible Electroporation Ablation System) pivotal trial, which evaluated the VARIPULSE variable-loop PFA catheter (Biosense Webster, Irvine, CA) integrated with electroanatomic mapping, demonstrated 100% acute pulmonary vein isolation, 97.5% first-pass isolation of targeted veins, 74.6% effectiveness at 12 months, and a primary adverse event rate of 2.9% [7]. Primary effectiveness across these and the persistent AF trials discussed below is summarized in Figure 2.

The trials summarized in Figure 2 differ in design, population, PFA platform, lesion set, monitoring intensity, and endpoint definition, and the results shown should not be read as head-to-head comparisons of relative efficacy. A 2026 meta-analysis of four randomized trials comparing PFA with thermal ablation found no significant difference in treatment success, serious adverse events, recurrent atrial arrhythmia, or repeat ablation, with most pooled outcomes rated low or very low certainty [8].

Persistent Atrial Fibrillation

Evidence in persistent AF has matured rapidly. In the PULSED AF pivotal trial, 1-year freedom from the composite endpoint of acute failure, arrhythmia recurrence, or antiarrhythmic escalation was 55.1% in the persistent AF cohort, with a primary safety event rate of only 0.7% and no cases of coronary spasm or phrenic, esophageal, or pulmonary vein injury [3]. The ADVANTAGE AF (A Prospective Single Arm Open Label Study of the FARAPULSE Pulsed Field Ablation System in Subjects with Persistent Atrial Fibrillation) trial, the first large prospective study combining PVI with posterior wall isolation using the pentaspline Farapulse catheter specifically in persistent AF, reported a primary effectiveness of 63.5% and a primary safety event rate of 2.3% at 1 year, reinforcing PFA as a viable first-line strategy for persistent AF rather than a paroxysmal-only technology [9]. The incremental benefit of adjunctive posterior wall isolation remains unsettled. ADVANTAGE AF and earlier cryoballoon-based trials suggested a favorable effect, but the randomized CORNERSTONE AF trial found no additional benefit of posterior wall isolation over pulmonary vein isolation alone in persistent and long-standing persistent AF [11]. This adjunctive strategy should therefore not yet be regarded as a settled component of the persistent AF ablation approach. These findings are complemented by the AVANT GUARD trial, a randomized comparison of PFA against antiarrhythmic drug therapy as first-line treatment in 310 patients with treatment-naive persistent AF, which found 12-month treatment success in 56.0% of the PFA group versus 30.1% of the drug-therapy group (hazard ratio for treatment failure 0.46, 95% CI 0.33-0.65), supporting PFA as an effective initial rhythm-control strategy rather than a second-line option reserved for drug failure [10].

Real-World Registries

Randomized trials are necessarily conducted under controlled conditions with selected operators; large multinational registries provide a complementary, real-world view. The MANIFEST-PF (Multi-National Survey on the Methods, Efficacy, and Safety on the Post-Approval Clinical Use of Pulsed Field Ablation) registry, encompassing the post-approval use of the pentaspline Farapulse catheter across 24 European centers and 77 operators, found no esophageal damage or pulmonary vein stenosis, with only a single case of persistent phrenic nerve palsy at 1 year [5]. The subsequent MANIFEST-17K study expanded this experience to 17,642 patients across 106 centers, the largest safety dataset for PFA to date [6]. Major complications occurred in approximately 1% of patients, with pericardial tamponade in 0.36% and vascular access complications in 0.30%; stroke occurred in only 0.12% of patients [6]. As with the earlier registry, no esophageal complications, pulmonary vein stenosis, or persistent phrenic nerve palsy were reported; transient phrenic nerve palsy occurred in 0.06% of patients [6]. Notably, stroke rates were lower in MANIFEST-17K than in MANIFEST-PF, an improvement attributed largely to more meticulous sheath management to prevent inadvertent air embolism, illustrating how procedural technique continues to evolve alongside the technology itself [5,6].

Safety profile and energy-specific adverse events

**Figure 3:**
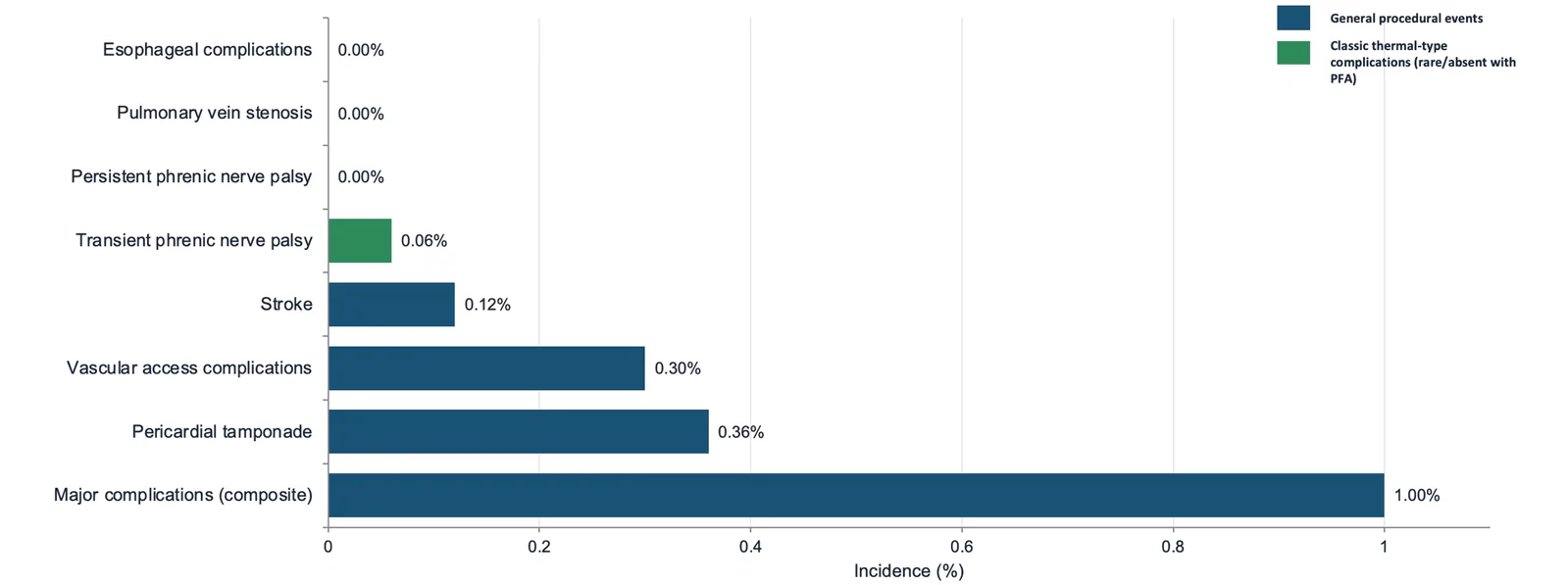
Safety outcomes in the MANIFEST-17K real-world registry (pulsed field ablation (PFA), N = 17,642 patients across 106 centers) Bars in navy represent general procedural complications; bars in green represent complications classically associated with thermal ablation energy sources, which were rare or not observed in this PFA cohort. Transient phrenic nerve palsy occurred in 0.06% of patients; no persistent phrenic nerve palsy, pulmonary vein stenosis, or esophageal complications were reported. Figure created by the authors using data from Ekanem et al., MANIFEST-17K registry [6].

The aggregate trial and registry data support a favorable overall safety profile for PFA, particularly regarding the thermal complications that have historically limited RF and cryoballoon ablation (Figure 3) [1,3,5-7,9]. However, PFA introduces its own energy-specific adverse-event spectrum that electrophysiologists must recognize.

Coronary artery vasospasm has emerged as a distinctive PFA-associated complication, particularly during ablation near the coronary ostia or cavotricuspid isthmus [2]. Proposed mechanisms include electroporation-mediated release of vasoconstrictive mediators from vascular endothelium and smooth muscle, autonomic nervous stimulation causing sympathetic-parasympathetic imbalance, and vasoconstrictive effects of free heme and oxyhemoglobin liberated by intravascular hemolysis [2,4]. Preclinical angiographic and histologic characterization studies in an animal coronary model suggest the incidence and severity of spasm may vary by PFA waveform, underscoring that not all PFA systems carry identical risk profiles [12].

Intravascular hemolysis is a second energy-specific phenomenon: high-voltage pulses generate transmembrane potentials in erythrocytes sufficient to cause pore formation and colloid-osmotic rupture [4]. While typically subclinical, severe hemolysis has been associated with acute kidney injury through heme-mediated proximal tubular damage, and current guidance emphasizes periprocedural hydration and monitoring of renal function and hemoglobinuria, particularly in patients receiving multiple applications or pre-existing renal impairment [4]. This risk is not uniform across platforms. A prospective comparison of three commercially available PFA catheters found laboratory-defined severe intravascular hemolysis in 37.0% of patients treated with a pentaspline catheter, compared with 26.1% and 14.7% of patients treated with circular-array and lattice-tip catheters, respectively; catheter type was an independent predictor of hemolysis risk [13]. Different PFA platforms and waveforms should therefore not be assumed interchangeable with respect to this complication. Related to this, microbubble formation from electrolysis, boiling, and degassing during pulse delivery has been identified as a potential contributor to silent cerebral embolism and is an active area of waveform and catheter engineering aimed at mitigation [4].

Conversely, complications characteristic of thermal ablation, atrioesophageal fistula, symptomatic pulmonary vein stenosis, and persistent phrenic nerve palsy, have shown a markedly reduced observed incidence across the large registries described above [5,6]. This shift in the risk profile, from thermal collateral injury toward a distinct set of electroporation-mediated effects that vary by platform and waveform, should inform periprocedural monitoring rather than be read simply as PFA being unconditionally "safer."

Comparison with thermal ablation modalities

Head-to-head data from ADVENT indicate that PFA is noninferior to thermal ablation for both efficacy and the composite primary safety endpoint in paroxysmal AF, with shorter procedure and ablation times but longer fluoroscopy exposure [1]. Procedural efficiency, a purported advantage of PFA given its point-and-shoot, single-catheter application without the need for extensive repositioning, has been reproduced across most registries, though this benefit is operator- and system-dependent [5,6]. From a safety-composition standpoint, PFA trades a small but real risk of coronary spasm and hemolysis-related renal injury, the magnitude of which varies by catheter platform and waveform [12,13], for a markedly reduced observed incidence of esophageal and pulmonary vein complications that have historically driven the most feared, if rare, outcomes of thermal ablation [1,2,4-6].

These individual-trial comparisons should be considered alongside pooled randomized evidence. A 2026 meta-analysis of four RCTs (1,294 patients, 653 receiving PFA) found no significant difference between PFA and thermal ablation in treatment success (risk ratio 1.11, 95% CI 0.87-1.42), serious adverse events (risk ratio 1.24, 95% CI 0.38-4.09), recurrent atrial arrhythmia (risk ratio 0.86, 95% CI 0.54-1.38), or repeat ablation (risk ratio 0.89, 95% CI 0.18-4.38), and procedural time was numerically shorter with PFA (mean difference -38.8 minutes; the meta-analysis authors reported this as significant at p=0.04, though the 95% CI, -75.5 to 2.1, itself spans zero, so this finding should be interpreted cautiously). Most pooled outcomes were graded as low or very low certainty, and larger, longer-term randomized trials are needed before firm comparative conclusions can be drawn [8].

Longer-term data are beginning to emerge: the ADVENT long-term outcomes (ADVENT-LTO) extension recently reported a 4-year treatment success of 72.8% with PFA versus 64.3% with thermal ablation, a difference that did not reach statistical significance, supporting durability of lesion sets created by this fundamentally different biophysical mechanism while underscoring that larger, longer-term studies are still needed [14]. The design, platform, comparator, and key results of the trials and registries discussed above are summarized in Table 1.

**Table 1:** Summary of major PFA trials and registries discussed in this review PFA, pulsed field ablation

Trial/Registry	Design	AF Type	PFA Platform	Comparator	Primary Endpoint	Key Result	Ref
ADVENT	RCT	Paroxysmal	Farapulse (pentaspline)	Thermal (RF/cryoballoon)	12-mo freedom from atrial arrhythmia	73.3% vs 71.3% (noninferior)	[1]
PULSED AF	Single-arm	Paroxysmal + persistent	PulseSelect (circular array)	Performance goal	1-yr composite freedom (persistent cohort)	55.1%; safety events 0.7%	[3]
AdmIRE	Single-arm	Paroxysmal	VARIPULSE (variable-loop)	Performance goal	12-mo effectiveness	74.6%; PAE 2.9%	[7]
ADVANTAGE AF	Single-arm	Persistent	Farapulse (pentaspline) + PWI	Performance goal	1-yr primary effectiveness	63.5%; PAE 2.3%	[9]
CORNERSTONE AF	RCT	Persistent / long-standing	RF or cryoballoon (non-PFA)	PVI alone (no PWI)	18-mo freedom from atrial arrhythmia	No incremental benefit of adjunctive PWI	[11]
AVANT GUARD	RCT	Persistent (treatment-naive)	PFA (platform not detailed)	Antiarrhythmic drug therapy	12-mo treatment success	56.0% vs 30.1%	[10]
MANIFEST-PF	Registry	Paroxysmal + persistent	Farapulse (pentaspline)	None	1-yr safety	No esophageal injury or PV stenosis; 1 persistent phrenic palsy	[5]
MANIFEST-17K	Registry	Paroxysmal + persistent	Farapulse (pentaspline)	None	Safety	Major complications ~1%; no esophageal injury, PV stenosis, or persistent phrenic palsy	[6]
ADVENT-LTO	RCT extension	Paroxysmal	Farapulse (pentaspline)	Thermal (RF/cryoballoon)	4-yr treatment success	72.8% vs 64.3% (NS)	[14]

Emerging applications and future directions

Beyond standard pulmonary vein isolation, PFA is being extended to several adjunct and investigational applications, including left atrial posterior wall isolation as an adjunct in persistent AF [9,11], cavotricuspid isthmus ablation for typical atrial flutter using focal-linear catheter designs, and ablation in patients with cardiac implantable electronic devices, in whom thermal energy poses additional lead-related concerns. Perhaps most notably, PFA is being investigated, off-label and outside current U.S. Food and Drug Administration-approved indications, as a treatment for ventricular arrhythmias [15]. In the largest published multicenter series to date, 80 patients with symptomatic outflow tract premature ventricular contractions were treated with a focal monopolar PFA catheter across four European centers; acute procedural success was achieved in all patients, and at a median follow-up of 8 months, clinical success was achieved in 88%, with PVC burden falling from a median of 21.4% before ablation to 0.0% at follow-up [16]. An earlier two-center series of 20 patients similarly reported no PVC recurrence in 85% of patients during in-hospital monitoring and complete PVC elimination in 60% at follow-up [17].

These results raise the possibility of a nonthermal alternative for ventricular substrates historically ablated with RF, including those near coronary arteries or the His-Purkinje system where thermal injury risk is a limiting factor [16,17]. These early results should not be extrapolated beyond the studied substrate. Ventricular myocardium is substantially thicker than atrial myocardium, and the lesion depth achievable with current PFA waveforms, optimized largely for thin-walled atrial tissue, has not been systematically characterized for thicker substrates such as scar-related ventricular tachycardia. Extending outflow-tract premature ventricular contraction findings to broader ventricular arrhythmia substrates is therefore premature until adequately powered, controlled trials are available [16,17].

Next-generation catheter platforms, including pentaspline, basket, variable-loop, and focal designs from multiple manufacturers, are diversifying the technical approaches to PFA delivery, each with distinct waveform characteristics that appear to influence both efficacy and the specific complication profile, for example, differential coronary spasm rates by waveform [12]. Integration of PFA catheters with electroanatomic mapping and lesion-index feedback, as demonstrated in AdmIRE, is likely to further refine procedural precision [7]. Comparative-effectiveness and mechanistic studies distinguishing among these platforms will be an important focus of the coming several years.

Limitations of current evidence

Several caveats temper enthusiasm for PFA. Most pivotal trials to date are single-arm or industry-sponsored studies with relatively short (one-year) follow-up; long-term lesion durability and late arrhythmia recurrence data remain limited, though multi-year extensions are beginning to emerge [1,3,7,9]. Pooled randomized evidence comparing PFA with thermal ablation also remains limited, with existing meta-analyses rating most outcomes as low or very low certainty [8]. Registry safety data, while reassuring in aggregate, are subject to reporting and selection biases inherent to postapproval, non-randomized designs; and complication rates for less common applications, such as ventricular ablation or posterior wall isolation, are based on small case series or single randomized trials with conflicting results rather than adequately powered trials with long-term follow-up [11,16,17]. Finally, because multiple PFA systems with distinct waveforms are now commercially available, safety and efficacy findings from one platform cannot be assumed to generalize to another, a point increasingly emphasized by waveform- and platform-specific spasm and hemolysis data [4,12,13].

## Conclusions

Pulsed field ablation has established itself, within a remarkably short interval, as a noninferior and, in several respects, complementary alternative to thermal catheter ablation for atrial fibrillation. Its tissue-selective, nonthermal mechanism has been associated with a markedly reduced observed incidence of the classic thermal complications of atrioesophageal fistula and pulmonary vein stenosis across large trial and registry populations, while introducing a distinct set of energy-specific risks, which vary meaningfully by catheter platform and waveform that require dedicated recognition and management strategies rather than being assumed away by the technology's novelty.

As indications expand beyond pulmonary vein isolation into posterior wall isolation, atrial flutter, and ventricular arrhythmias, and as longer-term, adequately powered comparative data accumulate, pulsed field ablation is positioned to become a foundational, rather than merely alternative, energy source in clinical cardiac electrophysiology, though results from individual trials should be interpreted within the context of each study's design and population rather than as direct cross-platform comparisons.
